# Common Peptides Study of Aminoacyl-tRNA Synthetases

**DOI:** 10.1371/journal.pone.0020361

**Published:** 2011-05-27

**Authors:** Assaf Gottlieb, Milana Frenkel-Morgenstern, Mark Safro, David Horn

**Affiliations:** 1 The Balvatnik School of Computer Science, Tel Aviv University, Tel Aviv, Israel; 2 Structural Biology and Biocomputing Programme, Spanish National Cancer Research Centre, Madrid, Spain; 3 Department of Structural Biology, Weizmann Institute of Science, Rehovot, Israel; 4 School of Physics and Astronomy, Tel Aviv University, Tel Aviv, Israel; Georgia Institute of Technology, United States of America

## Abstract

**Background:**

Aminoacyl tRNA synthetases (aaRSs) constitute an essential enzyme super-family, providing fidelity of the translation process of mRNA to proteins in living cells. They are common to all kingdoms and are of utmost importance to all organisms. It is thus of great interest to understand the evolutionary relationships among them and underline signature motifs defining their common domains.

**Results:**

We utilized the Common Peptides (CPs) framework, based on extracted deterministic motifs from all aaRSs, to study family-specific properties. We identified novel aaRS–class related signatures that may supplement the current classification methods and provide a basis for identifying functional regions specific to each aaRS class. We exploited the space spanned by the CPs in order to identify similarities between aaRS families that are not observed using sequence alignment methods, identifying different inter-aaRS associations across different kingdom of life. We explored the evolutionary history of the aaRS families and evolutionary origins of the mitochondrial aaRSs. Lastly, we showed that prevalent CPs significantly overlap known catalytic and binding sites, suggesting that they have meaningful functional roles, as well as identifying a motif shared between aaRSs and a the Biotin-[acetyl-CoA carboxylase] synthetase (birA) enzyme overlapping binding sites in both families.

**Conclusions:**

The study presents the multitude of ways to exploit the CP framework in order to extract meaningful patterns from the aaRS super-family. Specific CPs, discovered in this study, may play important roles in the functionality of these enzymes. We explored the evolutionary patterns in each aaRS family and tracked remote evolutionary links between these families.

## Introduction

The aminoacyl-tRNA synthetases (aaRSs) are key participants in the translation mechanism of the cell, catalyzing the esterification of specific amino acids and their corresponding tRNAs. Extensive studies have been carried out regarding their structures and functions [1], [2], [3], [4], [5], [6], [7], [8], [9]. Based on their three-dimensional structures, aaRS enzymes were divided into two nonequivalent classes. Although all aaRSs perform the same function of tRNA aminoacylation, the different structural topologies of their active sites and the character of signature sequences indicate that the two classes originate from different ancestors [10]. Their key role in the heart of the translation process and their connection to the genetic code make them candidates for evolutionary studies, aiming to pinpoint the way mRNA translation has evolved throughout evolution [11], [12], [13], [14], [15], [16]. Extensive evolutionary studies of aaRSs were carried out by Woese *et al.* [11] and Wolf *et al.* [13] a decade ago. They have shown that different aaRS families lead to different evolutionary tree structures, thus pointing out the evidence for lateral gene transfer among such families.

Here we studied the aaRS super-family using the Common Peptides (CPs) methodology. CPs are particular sets of deterministic motifs, composed of at least five consecutive amino-acids, selected by the Motive Extraction (MEX) algorithm (18). We exploited the CP framework for pursuing four objectives: (i) identify novel class-determining signature CPs, possibly bearing functional roles; (ii) elucidate remote homology among aaRSs and between aaRS and distant enzymes; (iii) uncover evolutionary patterns of aaRSs and (iv) identify CPs that reside on catalytic and binding sites. Class-specific CPs may be used to aid classification of novel aaRS sequences to these classes (analogously to enzyme classification of Kunik *et al.* [17]) as well as possibly signifying functional sites. CPs are also useful in determining relations between sequences when other sequence-similarity approaches (such as BLAST) reach their limits of applicability. Representing aaRS sequences in CP space (spanned by all CP motifs), we compared different aaRS families on the three kingdoms of life. The CP methodology has been successfully employed for olfactory receptor studies [18], demonstrating their possible utilization for evolutionary studies. Here we studied mainly the root of evolution, i.e. the highest ranks of the tree of life, as well as mitochondrial aaRS sequences, exhibiting mixed evidence for their evolutionary relationship to Bacteria and Eukarya. Last, we who that CPs significantly reside on catalytic and binding sites and derive the novel GIL[IVT]E motif, residing on both Threonyl tRNA synthetase (ThrRS) binding site as well as the structurally related Biotin-[acetyl-CoA carboxylase] synthetase (birA).

Thus we demonstrate that the CP methodology provides a new perspective of aaRSs class signatures and organization and can help to identify novel functional regions on aaRS sequences of different organisms.

## Results

### Frequent CPs

We studied 22 aaRS families, including 5,406 aaRS sequences (Table 1). We generated a list of 10,612 CPs by applying a two-stage algorithm: (i) Applying MEX [19] on each of the 22 aaRS families separately to extract family-associated CPs and (ii) merging the 22 CP lists and searching for occurrences of each CP in all aaRS families (see Methods).

**Table 1:** Properties of aaRS, ordered by class.

EC	Name	Class	# of sequences	# of MEX CPs	# of observed CPs	# of specific CPs
6.1.1.1	Tyrosyl tRNA synthetase (TyrRS)	I	261	400	758	239
6.1.1.2	Tryptophanyl tRNA synthetase (TrpRS)	I	121	163	323	102
6.1.1.4	Leucyl tRNA synthetase (LeuRS)	I	344	1031	1730	591
6.1.1.5	Isoleucyl tRNA synthetase (IleRS)	I	271	871	1608	568
6.1.1.9	Valyl tRNA synthetase (ValRS)	I	211	641	1293	378
6.1.1.10	Methionyl tRNA synthetase (MetRS)	I	248	634	1121	386
6.1.1.16	Cysteinyl tRNA synthetase (CysRS)	I	362	505	998	301
6.1.1.17	Glutamyl tRNA synthetase (GluRS)	I	373	645	1237	407
6.1.1.18	Glutaminyl tRNA synthetase (GlnRS)	I	37	96	178	50
6.1.1.19	Arginyl tRNA synthetase (ArgRS)	I	327	677	1275	421
6.1.1.3	Threonyl tRNA synthetase (ThrRS)	II	279	671	1128	431
6.1.1.6	Lysyl tRNA synthetase (LysRS)	II (I)*	192	340	651	175
6.1.1.7	Alanyl tRNA synthetase (AlaRS)	II	193	506	1019	314
6.1.1.11	Seryl tRNA synthetase (SerRS)	II	345	489	874	264
6.1.1.12	Aspartatyl tRNA synthetase (AspRS)	II	294	586	992	341
6.1.1.14	Glycyl tRNA synthetase (GlyRS)	II	226	432	773	265
6.1.1.15	Prolyl tRNA synthetase (ProRS)	II	369	792	1313	475
6.1.1.20	Phenylalanyl tRNA synthetase (PheRS)	II	495	682	1576	418
6.1.1.21	Histidyl tRNA synthetase (HisRS)	II	312	393	838	246
6.1.1.22	Asparaginyl tRNA synthetase (AsnRS)	II	124	213	402	130
6.1.1.n2	O-Phosphoseryl-tRNA synthetase (SepRS)	II	17	31	87	20
6.1.1.26	Pyrrolysyl tRNA synthetase (PylRS)	II	5	16	20	11

*Some of the LysRS sequences are class I.

CPs represent conserved deterministic sequence-motifs, where higher abundance signifies higher conservation. Since regions of sequence conservation are frequently associated with structural motifs and functional sites, we are interested in the most abundant CPs (Table 2). All top occurring CPs are found exclusively in class I sequences.

**Table 2:** Top 10 most frequent CPs.

CP	sequence occurrences (percentage)	# of aaRSs	Bacteria (percentage)	Eukarya (percentage)	Archaea (percentage)	mitochondria (percentage)
KMSKS	1364 (25%)	9	1196 (26.4%)	24 (14.5%)	102 (17.1%)	42 (39.6%)
KSLGN	502 (9%)	9	440 (9.7%)	5 (3.0%)	40 (6.7%)	17 (16.0%)
ISRQR	345 (6%)	3	296 (6.5%)	0 (0.0%)	36 (6.0%)	13 (12.3%)
GRPGWH	333 (6%)	1	297 (6.5%)	6 (3.6%)	24 (4.0%)	6 (5.7%)
PSPTG	329 (6%)	2	319 (7.0%)	3 (1.8%)	0 (0.0%)	7 (6.6%)
FPHHE	327 (6%)	1	294 (6.5%)	0 (0.0%)	28 (4.7%)	5 (4.7%)
PYANG	318 (6%)	2	295 (6.5%)	0 (0.0%)	18 (3.0%)	5 (4.7%)
RQRYWG	310 (6%)	2	283 (6.2%)	0 (0.0%)	22 (3.7%)	5 (4.7%)
SKSKG	299 (6%)	8	266 (5.9%)	1 (0.6%)	32 (5.4%)	0 (0.0%)
PYPSG	294 (5%)	2	284 (6.3%)	0 (0.0%)	4 (0.7%)	6 (5.7%)

The first CP in Table 2, KMSKS, is one of two well-known signatures of class I. This motif is involved in catalysis of the second step of the aminoacylation reaction [20], [21]. The second well-known signature, HIGH, is not found in this list because our MEX CP search was limited to CPs of length five or more amino acids.

### CPs as Class and Sub-Class Signatures

**Figure 1:**
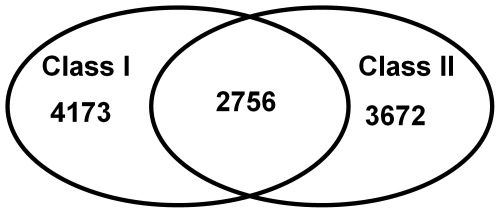
Relative abundance of CPs in class I and class II synthetases. Venn diagram showing relative abundance of CPs in class I and class II synthetases.

CPs are generally nonspecific to a particular aaRS family, but some of them are prevalent in either class I or class II synthetases (Figure 1).

Table 3 lists novel CPs that preferentially occur in only one class and appear in at least six different aaRS families of that class, specifying the number of occurrences of each CP in distinct proteins and aaRS families. CPs that contain known class I signature motifs (HIGH and KMSKS) were omitted from this list.

**Table 3:** Novel class-specific CPs.

CP	# of class I aaRSs	# of class I occurrences	# of class II aaRSs	# of class II occurrences
TADEI**	8	47	1	1
ALADE	8	37	1	2
KSLGN	7	500	2	2
SKSKG	7	299	1	0
SKGNV	7	182	1	0
DVIAR**	7	73	0	0
DVVAR**	7	60	1	2
ADAIR	7	39	1	0
GLDLL	7	35	1	1
GVERL* †	0	0	8	92
DLVEE*	1	3	7	66
GLDRI*	1	1	7	43
AEAVL	1	2	7	24
ERISA*	0	0	7	24
LRLAE**	0	0	6	38
AAGVR	2	2	6	47

*Overlap a binding site.

**less than four residues apart from a binding site.

†GVERL may be part of more general class II motifs [3], [22].

The novel CPs appearing preferentially in class I or II may be used to aid classification of novel aaRS sequences to these classes (in analogy to the use made in classifying enzymes by [17]). Furthermore, these CPs may signify a functional or structural constrained region, related to the specific type of operation carried out by each class. We extracted nine CPs specific to class I that can augment the known KMSKS and HIGH signatures and seven CPs which can be regarded as novel class II signatures. No deterministic motifs are currently known for class II [3], [22]. We found that four of the signatures overlap known binding sites: GVERL overlaps binding site of 4-Amino-1,4-Dioxobutan-2-Aminium Adenosine- 5′-Monophosphate (PDB ID 1X54 [23]), DLVEE overlaps Magnesium ion (PDB ID 2AKW [7]) and GLDRI and ERISA overlap ATP binding sites (PDB IDs 1YFR [24] and 1ASZ [25], where the CPs are slightly altered to GLERV and ERIAS, correspondingly). In addition, three signatures are a distance of 4 residues from Zinc ion (TADEI), tRNA (LRLAE) and ((2r,3s,4r,5r)-5-(6-amino-9h-purin-9-yl)-3,4- dihydroxytetrahydro-2-furanyl)methyl sulfamate (LMS) (the merged motif of DV[VIL]R).

It is of particular interest to look at the following four class I aaRSs: Valyl tRNA synthetase (ValRS), Isoleucyl tRNA synthetase (IleRS), Leucyl tRNA synthetase (LeuRS) and Methionyl tRNA synthetase (MetRS). According to Woese *et al.* [12], they originate from a common ancestor. To test this assumption we searched for CPs that appear on all these four aaRS families, and do not appear anywhere else in our data. We extracted a set of 11 CPs specific to these four aaRSs, interestingly occurring only in bacterial or mitochondrial sequences (Table 4). Comparing this result to alternative random sets of four class I aaRSs, we found no random set exceeding four shared CPs.

**Table 4:** CP frequency in class I aaRS families predicted to share the same origin.

CP	LeuRS	IleRS	ValRS	MetRS	sum
GNVISP	2	11	2	1	16
AEELW	59	1	3	1	64
GKNVL	44	4	11	2	61
HRMRG	3	1	15	1	20
KRMQG	42	2	31	3	78
LYNKG	5	4	14	2	25
NTVDP	24	1	3	2	30
NVVDP	45	32	4	24	105
RMQGY	40	3	20	4	67
RYHRM	9	4	25	2	40
RYKRM	24	3	27	4	58

It should be noted that out of the 11 CPs in Table 4, two may be joined into the regular expression motif N[T/V]VDP, with 135 hits, which immediately follows the KMSKS class I signature and four that can be joined into the motif RY[H/K]RM[R/Q]G with 98 occurrences. By this we conclude that the CP analysis indicates indeed that there exists some important relationship between these four aaRS families.

### aaRS similarities using CPs as features

CPs span a feature space in which the aaRS sequences are represented. Summing the occurrences of each CP over all aaRS family sequences, we obtained family-specific CP-based features. We next computed Pearson correlations between all pairs of aaRS families to obtain inter-aaRS similarity measures, correcting for CP overlaps across sequences (Methods). While the absolute values of the correlations are small, some correlations proved to be above the background. We constructed a background model by randomly shuffling the CP assignments to each aaRS family while retaining the overall CP family coverage (Methods), retaining correlations corresponding to p-value<0.01. Three pairs of class I aaRSs obtain significant correlations: TrpRS is significantly similar to TyrRS, LeuRS and CysRS (Figure S1). Due to low similarity between the 20 standard aaRS families and either O-Phosphoseryl-tRNA synthetases (SepRS) or Pyrrolysyl tRNA synthetases (PylRS), we removed the latter two enzyme families from the analysis.

A striking difference in inter-aaRS similarity emerges when the correlations are calculated separately for each kingdom (mitochondria considered as a separate kingdom). Bacteria sequences comprise the majority of aaRSs sequences, resulting in similar inter-aaRS correlations (Figure S2). Correlations between eukaryotic enzymes are mostly higher than observed in other kingdoms (0.29 between GluRS and GlnRS and 0.57 between ProRS and GluRS) (Figure S3). Additionally, similarities between (class I) MetRS and TyrRS stand out above the background. Indeed, Siatecka *et al.* [26] noted that both MetRS and TyrRS in various species share EMAP II-like domains. Additionally, correlations are evident between class II enzymes AspRS and LysRS, which were reported to share similar ‘basic faced’ *α*-helix (BFAH) structural features [27]. Interestingly, (class II) ProRS is correlated with (class I) GluRs and GlnRS. The high correlation between eukaryotic ProRS and GluRS is in line with the observation made by [28], [29], pointing out that in Bacteria and Archaea, distinct genes encode the two proteins while in several organisms from the eukaryotic phylum of coelomate metazoans, the two polypeptides are carried by a single polypeptide chain to form a bifunctional protein, postulated to result from a gene fusion event. Accordingly, the correlation between ProRS and GlnRS stems from the similarity between GlnRS and GluRS.

Correlations between TrpRS and TyrRS in Archaea are viewed also in Bacteria, but correlations between ValRS and IleRS are not apparent (Figure S4). These two aaRSs were reported to have some similarity in sequence [12], [13]. In archaeal class II enzymes we found correlations between ProRS and GlyRS, which were reported to share a small domain that is predicted to possess an α-helical, coiled-coil structure [13].

Last, correlations between mitochondrial aaRSs exhibit a similar class I pattern as Bacteria with two exceptions: the emergence of correlation between TrpRS and IleRS, and the absence of correlation between TrpRS and TyrRS. In class II enzymes we observe correlations between PheRS and HisRS (Figure S5).

**Figure 2:**
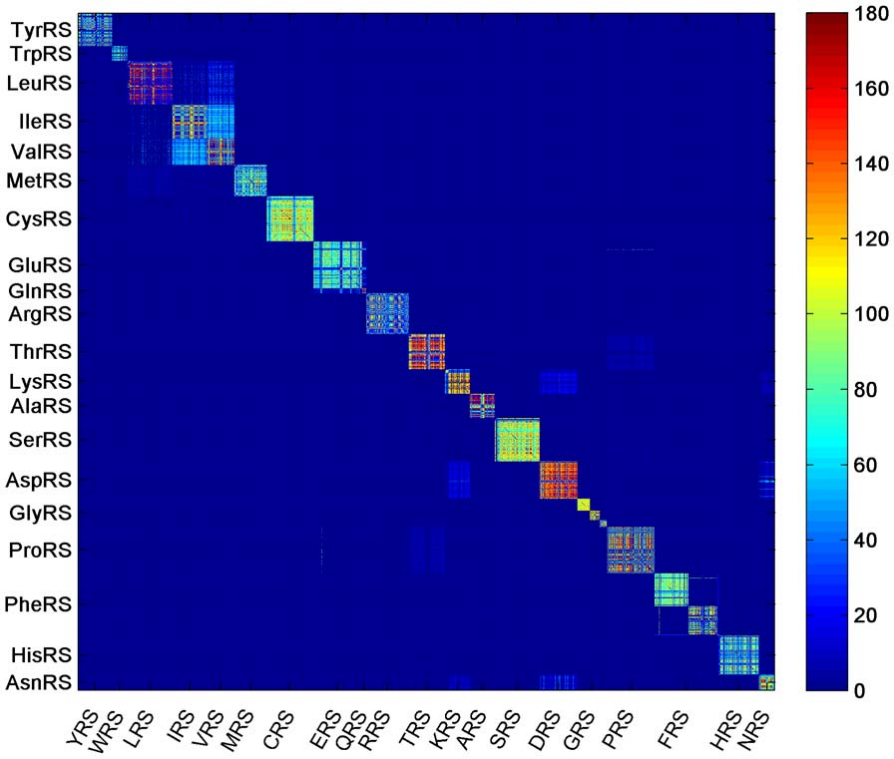
BLAST similarities between different aaRSs (log-scaled). E-values>0.01 are in blue.

Comparison with BLAST similarities reveals that many of the observed CP-space inter-aaRSs connections are not apparent using BLAST similarities with e-values below 0.01 or even as high as 1 (Figure 2). Specifically, the most prominent BLAST similarity between class I enzymes is between IleRS and ValRS, and to a lesser extent to LeuRS. However, the CP-similarities between class I aaRSs to TrpRS are not evident in BLAST similarities. In addition, most class II similarities evident in the CP representation are not evident using BLAST, including also the inter-class correlations of ProRS. Similarities in CP-space that are not reflected by BLAST may be explained by remote ancestry and by remnants of small conserved areas while BLAST similarities not reflected in CP-space may be due to existence of regions characterized by inexact amino acid resemblance that is not picked up by CPs. In order to validate that the difference between the CPs and BLAST similarities is not biased by matching variable sequence regions, we computed BLAST similarities on sequence domains of PFAM [30] and PROSITE [31] (Methods). PROSITE class I similarities are sparse since most class I PROSITE domains are based solely on the regions of KMSKS and HIGH signatures. Comparing aaRS families using domain similarity did not alter the results (Figures S6 and S7). Thus we observe that CP correlations carry complementary information to BLAST similarity.

### Evolutionary aspects of CPs

It has been demonstrated by [18] that reconstructing CPs onto a phylogenetic tree can track interesting evolutionary events. Following the same philosophy, we first examine the assignment of CPs to the different kingdoms of life (Mitochondria separated from Eukaryotes). Table 5 lists the relative abundance of CPs in each kingdom, showing the percentage of the CPs out of the full list that appear in each kingdom and the percentage of CPs appearing in each kingdom that are unique to this kingdom.

**Table 5:** CP Statistics for each kingdom of life (Mitochondria separated from the Eukaryotes).

Kingdom	# of proteins	Percentage of CPs	Specific CPs out of all observed in kingdom
Bacteria	4538	94.7%	62.8%
Eukarya	166	13.1%	3.7%
Archaea	596	28.9%	13.5%
Mitochondria	106	11.1%	1.3%

**Figure 3:**
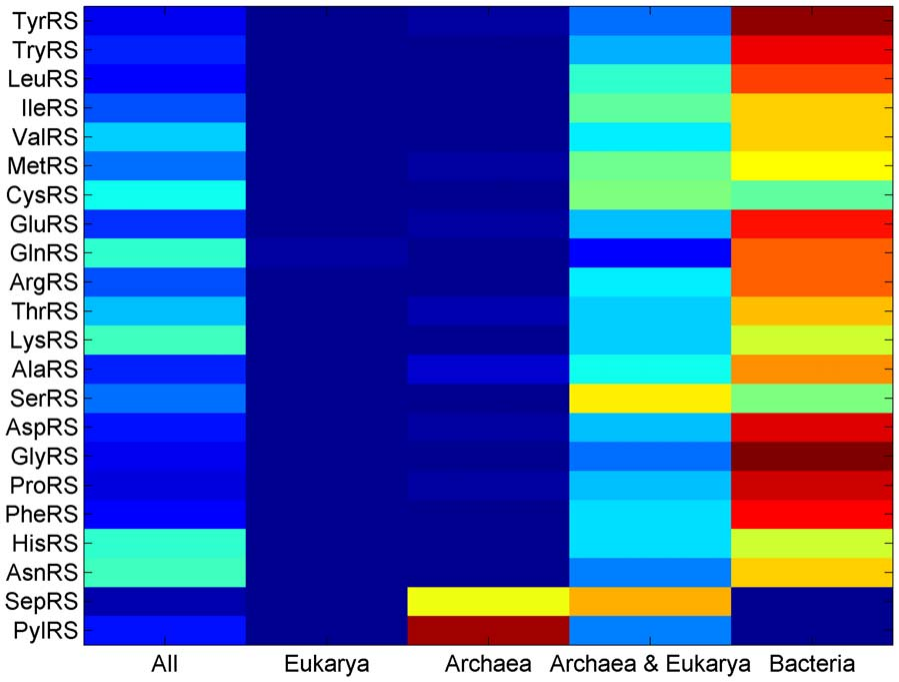
Distribution of different aaRS families across kingdoms according to the CP origin. Distribution of different aaRS families according to the CP origins: appearing in all 3 kingdoms, only in Eukarya, only in Archaea, Archaea and Eukarya together, and only in Bacteria.

In Ciccarelli's Tree of Life (ToL) [32], the tree assumes a topology in which Bacteria is an outgroup of Archaea and Eukarya. Accordingly, we defined five distinct sets of CPs, appearing in (i) all three kingdoms; (ii) the joint node of Archaea and Eukaryotes and (iii) each of the 3 kingdoms exclusively. Figure 3 displays the distribution of each CP set among the different aaRS families, enabling us to study the history of aaRS formation.

Since the majority of sequences are of bacterial origin, the majority of CPs are specific to the Bacteria lineage. Noted exceptions are the Seryl-tRNA synthetases (SerRS), with the majority of its CPs originating in the common ancestor of Archaea and Eukaryotes and CysRS, having comparable number of CPs originating in Bacteria and in the ancestor of Archaea and Eukarya. Both SerRS and CysRS exceptions are consistent with the observations of Woese *et al.* and Wolf *et al.* [12], [13], noting that their complex evolutionary pattern is not in line with the canonical form and is generally hard to interpret. As expected, PylRS CPs originate mainly in Archaea, but SepRS CPs originate almost equally in Archaea and the common ancestor of Archaea and Eukarya. It was hypothesized that SepRS is derived from PheRS and their common ancestor is conceivable as a homotetramer [9]. From Figure 3, however, it follows that their origins differ significantly. Supplementary Figure S8 displays finer details by excluding Bacteria-specific CPs from Figure 3 and excluding PylRS and SepRS for clarity.

### Mitochondria and CPs

Mitochondria are highly unusual organelles. They contain their own genetic material and protein-making machinery enwrapped in a double membrane. Mitochondria reside in eukaryotic cells, yet bear similarity to Bacteria in general and to α-Proteobacteria in particular [33], [34]. Various endosymbiotic models imply transfers of bacterial genes into the nuclear genome of the eukaryotic host [35]. Mitochondrial aaRSs reside in Eukaryotic nuclear DNA, however their origin remains largely unresolved [36]. We exploited the CPs in order to characterize the relations between mitochondrial aaRSs and the three kingdoms of life. For each aaRS family, we tested for enrichment of kingdom-specific CPs within mitochondrial CPs (Methods). We found 16 mitochondrial aaRSs that are enriched in Bacteria (AlaRS, ArgRS, AsnRS, AspRS, GluRS, GlyRS, IleRS, LeuRS, MetRS, PheRS, ProRS, SerRS, ThrRS, TrpRS, TyrRS and ValRS) (false discovery rate (FDR) <0.01)). Further focusing on α-Proteobacteria–specific CPs, we obtain similar results to Bacteria-specific CPs, with the exception of ArgRS, AsnRS and SerRS, which are not enriched in α-Proteobacteria (Methods). In contrast, only four mitochondrial aaRSs are enriched in Eukaryotes: GlyRS, HisRS, ThrRS and ValRS (FDR<0.01) and none are enriched within Archaeae. Notably, CysRS and LysRS are not enriched in both kingdoms, sharing very small numbers of CPs with Bacteria-specific CPs (5 and 3 shared CPs, respectively) and none with Eukaryotic-specific CPs. Mitochondrial GlnRS has no representation in our data since mitochondrial GlnRSs are absent from most Eukaryotes [37].

We found good agreement between our findings and the phylogenetic analysis of Brindefalk *et al.* [36]. They identified twelve aaRSs that have monophyletic grouping with α-Proteobacteria, out of which eleven are enriched in Bacteria and nine in α-Proteobacteria, exceptions including CysRS that is not enriched in Bacteria and additionally AsnRS and SerRS that are not enriched in α-Proteobacteria. A possible reason for the CysRS exception resides in the fact that CysRS includes only one nuclear gene, suggesting loss and replacement of either the mitochondrial or cytoplasmic CysRS. While Brindefalk *et al.* postulated that the mitochondrial CysRS was retained, it follows from our analysis that even if this assumption is correct, the bacterial signal has eroded, suggesting a relatively higher mutation rate. Regarding the AsnRS and SerRS exceptions, indeed Brindefalk *et al.* reported that neither displayed characteristic bacterial or cytoplasmic patterns. However, while the CPs could not trace the α-Proteobacteria signal, we could still find remnants of the remote ancestry of Bacteria.

Finally, eight aaRSs are reported to have a single eukaryotic aaRS nuclear gene in animals and fungi [36]. Based on the CP enrichment, we assessed which gene was lost in five cases (cytoplasmic or mitochondrial), excluding CysRS, GlnRS and LysRS that were not enriched in both kingdoms. Two of the single-gene mitochondrial aaRSs show enrichment in a single kingdom (AlaRS in Bacteria and HisRS in Eukaryotes), where AlaRS had the same assignment in [36] and HisRS assignment is consistent with the postulation made in [13]. For the other three (GlyRS, ThrRS and ValRS), we found enrichment in both Bacteria ad Eukarya. While we cannot infer in this case which aaRS gene was retained (the cytoplasmic or the mitochondrial), the fact that we observe both Bacteria-specific and Eukaryotic-specific CPs in the same mitochondrial sequences of these aaRSs suggests that interchanging of domains between the cytoplasmic and the mitochondrial genes might have occurred prior to the loss of one of them.

15 CPs are found to be specific to Mitochondria (Table 6). It is interesting to note that the low complexity CP “QQQQQ” that appears only in Mitochondria, appears in IleRS, LeuRS and HisRS. This CP usually appears more than once in a sequence and typically it is part of a longer stretch of Glutamines. This may point out that these proteins contain intrinsically unstructured regions (IURs) [38], [39].

**Table 6:** CPs specific to the Mitochondria.

CP	# of enzymes appears in
TTPIFYVN	9
SLESGH	7
VHSHW	7
ELADALGGLLNRCTA	5
QWGNYFLH	5
STWELLD	5
KIQQAA	5
CVRQTNGFVQRHAPWKL	4
ITNCGSGF	4
YKALEAVS	4
GTLLQPV	4
KLPEFNR	4
AVQHFW	4
QQQQQ	4
VLQWL	4

### Functional role of aaRSs

We looked for occurrences of our CPs in the protein sequences whose structures are deposited in Protein Data Bank (PDB) database [40]. We selected CPs which are present in more than half of the sequences of at least one aaRS, resulting in 50 prevalent CPs. 29 (58%) of the prevalent CPs cover known catalytic and binding sites (hypergeometric p<E^−8^, see Methods). Eight additional CPs are located at most three residues from a binding site (p<1.5E^−10^). The known class I signature KMSKS and its modified motif KLSKR corresponding to the catalytic site were omitted from the analysis. The majority of the binding sites hit by the prevalent CPs are related to the esterification of the corresponding amino acids, including the intermediates of the aminoacylation reaction (e.g. Tryptophanyl-5′AMP – TrpAMP) or their non-hydrolyzable analogs (e.g. 5′-O-[N-(L-tyrosyl)sulphamoyl]adenosine - Tyr-AMS) and also adenosine triphosphate (ATP), while two CPs cover the binding domain to tRNA. Additionally, three CPs hit binding sites of Chloride and Magnesium ions (Table S1). We thus see that the location of CPs is indeed highly associated with functional areas on the proteins.

### Biotin-[acetyl-CoA carboxylase] synthetase (birA) and aaRSs

BirA is a bi-functional protein, that acts as biotin-synthetase regulating its own transcription via binding to corresponding DNA regions. Structural similarity between its active sites and class II aaRS has been reported [41], [42], although structure-derived sequence alignment of BirA and class II aaRSs shows no clear sequence homology. It is thus of interest to find whether certain CPs derived from aaRSs are common also to birA, revealing local similarities that may relate to the structural similarity in the binding sites.

In order to select dominant CPs, common to both aaRSs and birA, we first performed the same procedure used for aaRSs on birA sequences, obtaining set of 1630 non-redundant birA CPs of length five or more. We selected CPs that appear in both aaRSs and birA lists, either by exact match or by full inclusion (i.e. birA CPs could be part of a larger aaRS CP and vice-versa), leading to a list of 28 common CPs (hypergeometric test, p<2.3E^−5^). We next retained four CPs prevalent in both aaRS and birA protein families, requiring appearance of each CP in a minimum of 20 sequences in both aaRSs and birA (Table 7). We note that across 20 random shuffling of the birA sequences, MEX extracted at most one CP, covering at most 4 sequences.

**Table 7:** Frequent CPs common to aaRSs and birA.

CP (alternatives)	Structural properties	# of birA occurrences	# of aaRSs occurrences	# of class I occurrences	# of class II occurrences
GILIE(GILVEGILTE)	Biotin binding site in birA,ThrAMS binding site in ThrRS	198	2	2	113
GALRL(GALLL)	Binding site for 4AD in AspRS	32	1	0	42
GEALG(GETLG)	Helix-turn-Helix in birA	22	4	1	31
LRAAL	α-helix in birA	86	13	43	7

*Alternatives in brackets mark one-mutation-distant CPs that were selected on birA but not in aaRSs.

4AD = 4-Amino-1,4-Dioxobutan-2-Aminium Adenosine- 5′-Monophosphate.

The most prevalent CP is GILIE (appearing in birA and aaRSs also as GILVE or GILTE). It covers more than a hundred sequences from both aaRSs and birA, occurring mostly in ThrRS (131 out of 140 sequences in our dataset). Interestingly, the motif GIL[IVT]E resides on the binding site of biotin in birA (PDB IDs 1HXD [43]) and resides in the close vicinity (2 residues apart) from the binding site of the non-hydrolyzable analog Thr-AMS region in ThrRS (PDB ID 1KOG [44]). Postulating that divergent evolution might have retained higher sequence similarity between aaRSs and birA, these common CPs may hint to a convergent evolution scenario rather than divergent evolution of the aaRSs and birA.

## Discussion

We have employed the Common Peptides (CPs) methodology to carry out an analysis of aminoacyl tRNA synthetases (aaRSs), identifying novel class-determining signature CPs, elucidating remote homology between aaRSs and between aaRS and the birA enzyme family and uncovering evolutionary patterns of aaRSs and particularly in Mitochondria. We further showed that prevalent CPs are significantly associated with enzyme functional sites.

We identified novel class I and class II signature motifs, that may aid future identification of these classes in newly found enzymes or Metagenomic data in an analogous way to [45]. Further research examining functional and structural roles of these signatures is required.

Using the CPs as a feature space in which aaRSs are expressed, we were able to identify similarities between different aaRS amino acid sequences. Correlations, calculated on sequences belonging to species from each kingdom separately, reveal differences in correlation patterns unique to each kingdom. Comparison of the CP based similarities to ones calculated by BLAST sequence alignment show that CP-space presents a complementary view, providing inter-aaRS connections that are not reflected by conventional sequence alignment. In particular, some inter-aaRS similarities, e.g. class I LeuRS and TrpRS or similarity between class I ProRS and GluRS (the latter is based on a similarity in a specific domain), were unobservable in the BLAST similarity plot while other inter-aaRS similarities elucidated via the CP framework were observed only in few sequences from each aaRS family, making the identification a hard task.

As shown in [18], CPs are particularly useful in tracing distant evolutionary origins. We have identified that CysRS and SerRS show distinctive evolutionary patterns and were able to infer relationships between mitochondrial aaRSs and other kingdom of life.

Last, we showed that prevalent CPs significantly overlap regions associated with catalytic activity and substrate binding in aaRSs with known 3D-structures, as well as identifying a motif that is common to the ThrRS and birA binding sites.

In summary, CP analysis provides a complementary view on aaRS enzymes, their relationships with each other and their evolution, as well as possible relations with other proteins that are not included in aaRS families. Their overlap with functional sites on the enzymes emphasizes their biological importance and makes them candidates for further analysis that, focusing on them, may lead to novel insights on the mechanism and evolution of aaRS enzymes..

## Materials and Methods

### Data

We downloaded 5,406 protein sequences belonging to 22 enzyme families of aminoacyl tRNA synthetases (Enzyme Commission (EC) number 6.1.1.x) from the Enzyme and UniProt databases [46], [47], [48]. aaRSs for the 20 standard amino acids (AA), pyrrolysine (PylRS) and O-phosphoseryl-tRNA synthetase (SepRS) were included in this study (Table 1).

1664 Biotin-[acetyl-CoA carboxylase] synthetases (birA) sequences were obtained from UniProt [47], [48].

Binding sites were downloaded from PDB [40].

### Method of Common Peptides

The datasets contain some highly similar enzyme sequences, belonging either to species of the same genus or different strains of the same species. In order to reduce redundancy we identified the groups of highly similar enzymes. This redundancy reduction was performed by first calculating BLAST pairwise sequence similarity [49] between all pairs. We selected a threshold of 90% sequence identity followed by single linkage clustering to create groups of highly similar proteins. Manual inspection of these sequences revealed that this threshold indeed clustered together sequences belonging to different strains of the same species or of the same genus, while lower thresholds clustered more remote homology pairs. Only one representative from each such group of redundant sequences was considered in this study. It was chosen according to the maximal average sequence identity to other group members. The remaining sequences thus represent a ‘non-redundant set’ of the Enzyme database for the aminoacyl tRNA synthetases.

In order to create a unified CP-space in which all sequences were represented, we followed the procedure described by Gottlieb *et al.* [18]. This procedure starts by applying the unsupervised Motif EXtraction algorithm (MEX) [19], [50] to each of the 22 non-redundant sets of enzyme sequences, resulting in 22 separate sets of Common Peptides (CPs), of length five amino-acids or more. The separate lists of CPs are then unified, decreasing redundancy from the unified list by removing long CPs that fully contain smaller CPs. The unified list contained 10,612 CPs. Finally, all CPs are searched on all aaRS sequences (including those from which the CP was not extracted by MEX) to construct a denser metric. We thus end up with a CP space, where each CP forms a feature and each sequence is sparsely represented by the occurrence of CPs on it.

### Calculating BLAST similarities and CP correlations

We used the Blastpgp program to find similarities between any aaRS sequence to all other aaRSs. Testing sequence similarities for E-values ranging from E^−5^ to 1 produced similar results. Computing BLAST similarities between domains, we downloaded PFAM [30] and PROSITE [31] domains from the Interpro database [51]. We extracted from each protein its corresponding domains and performed a BLAST sequence similarity alignment between these domains. Full sequence was retained for proteins lacking domain information (1 protein for PFAM and 3609 for PROSITE).

The Pearson correlation between different aaRSs were calculated based on the number of appearances of each CP on each aaRS sequences, normalized by the number of sequences of that aaRS. Some of the CPs overlap on some of the sequences (if two CPs overlap on a statistically significant portion of sequences, MEX would identify only the joined CP). In order to decouple the appearances of two overlapping CPs, we created a merged CP whenever two or more CPs overlap on a certain sequence, resulting in additional 20,391 concatenated CPs. CP occurrences were thus counted for each CP only on sequences where it did not overlap any other CP, whereas concatenated CPs were counted when overlapping CPs were identified on a sequence. Correlations were thus calculated on the joint 31,003 features, including both 10,612 CPs and 20,391 merged CPs. A random model was constructed by shuffling 100 times the assignments of the CPs to different aaRSs, retaining the same number of appearances for each CP.

### Assignment of proteins to kingdoms

The linking of species to the different kingdoms of life (Archaea, Bacteria and Eukarya), was utilized by using the Kyoto Encyclopedia of Genes and Genomes (KEGG) [52], [53], [54], European Bioinformatics Institute's (EBI) Karyn's Genomes [55] and Ciccarelli tree of life [32]. In addition, we separated mitochondrial sequences into a fourth group.

Full names of α-Proteobacteria were downloaded from [56]. We identified α-Proteobacteria sequences by matching only family name of the species (e.g. *Rickettsia*, *Brucella etc.*), resulting in 38 different α-Proteobacteria species.

### Enrichment of Mitochondrial aaRSs in kingdoms of life

For each aaRS family, we extracted the list of CPs that hit only one of the three kingdoms of life (Bacteria, Eukarya and Archaea) in that aaRS family, creating a list of CPs that are both aaRS- specific and kingdom-specific (but may occur in other kingdoms for other aaRSs). We next computed the hypergeometric enrichment of the CPs hitting the corresponding mitochondrial aaRS family in each of the kingdom-specific lists, correcting for false discovery rate (FDR) of 0.01.

### Identifying CP hits of binding and catalytic sites

We identified 50 prevalent CPs, present in more than half of the sequences of at least one aaRS (additions obvious two CPs - KMSKS and KLSKR CPs, corresponding to class I catalytic sites were omitted). From each aaRS family that is covered by these prevalent CPs, we selected the highest resolution PDB entry and looked for inclusion of the binding site in the CP, resulting in 29 exact hits. In order to identify additional eight CPs that reside in the vicinity of the biding site, we employed a looser threshold, requiring a maximal distance of three residues between the CP and the site.

## Supporting Information

Figure S1 Pearson cross-correlations of different aaRSs according to their shared CPs. Self correlations were left out for the purpose of clearer presentation.
(TIF)

Figure S2 Heat map of Pearson cross-correlations of different aaRSs according to their shared CPs in Bacteria. Self correlations were left out for the purpose of clearer presentation.
(TIF)

Figure S3 Heat map of Pearson cross-correlations of different aaRSs according to their shared CPs in Eukarya. Self correlations were left out for the purpose of clearer presentation.
(TIF)

Figure S4 Heat map of Pearson cross-correlations of different aaRSs according to their shared CPs in Archaea. Self correlations were left out for the purpose of clearer presentation.
(TIF)

Figure S5 Heat map of Pearson cross-correlations of different aaRSs according to their shared CPs in Mitochondria. Self correlations were left out for the purpose of clearer presentation.
(TIF)

Figure S6 BLAST Similarities between different aaRSs using PFAM domains. E-values> 0.01 are in blue.
(TIF)

Figure S7 BLAST Similarities between different aaRSs using PROSITE domains. E-values>0.01 are in blue.
(TIF)

Figure S8 Distribution of different aaRS according to the CPs appearing in all 3 kingdoms together (All), in Bacteria and Eukarya together (excluding Archaea) and in Eukarya and Archaea kingdoms exclusively.
(TIF)

Table S1 Occurrence of frequent CPs in aaRSs on sequences with a resolved structure.
(PDF)
