# Supplementary figures and images for: Common Peptides Study of Aminoacyl-tRNA Synthetases
Source: PLoS One. 2011 May 27;6(5):e20361. doi: 10.1371/journal.pone.0020361 (PMC3103580; doi:10.1371/journal.pone.0020361)

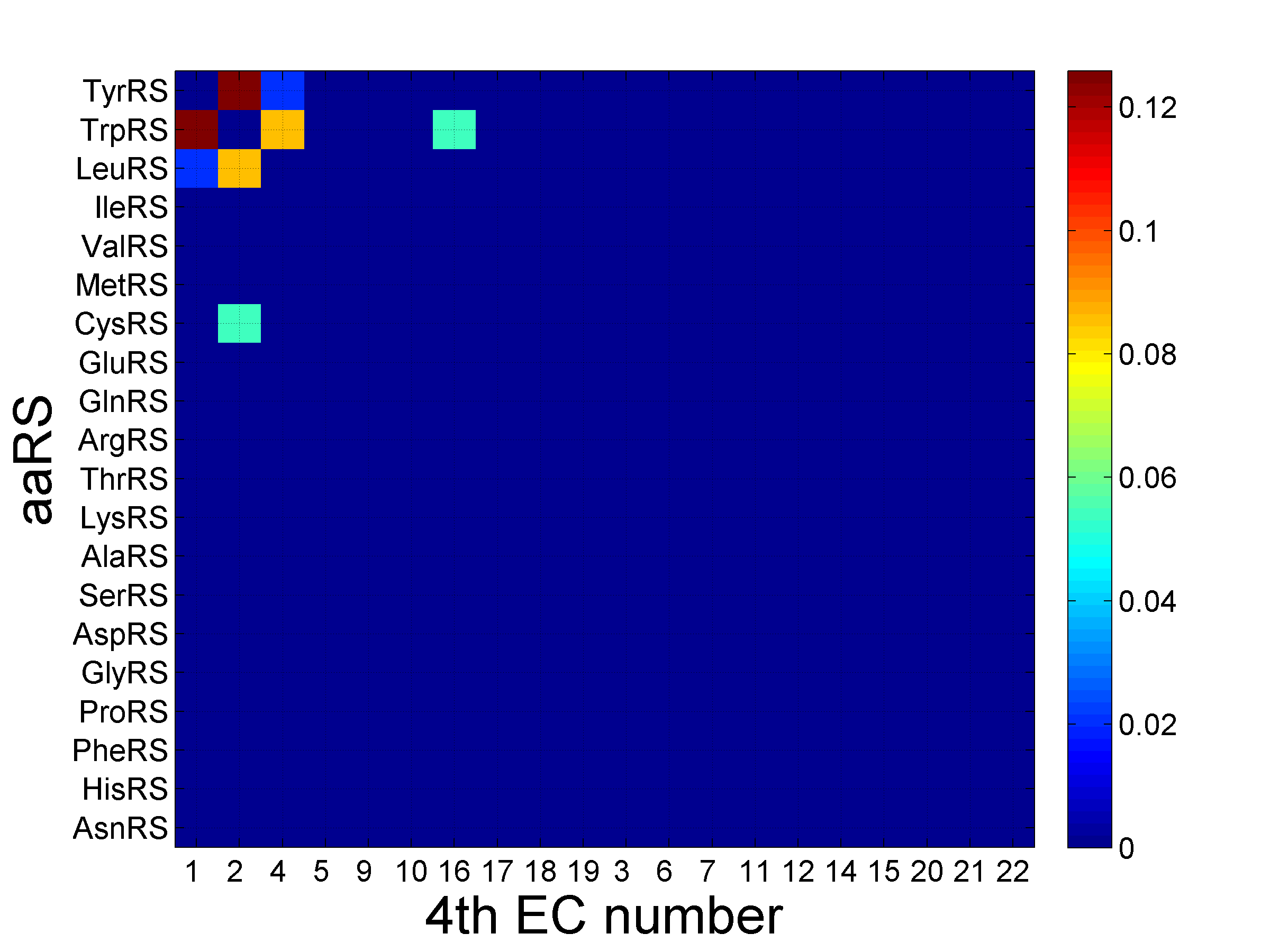

Supplement: Figure S1 — Pearson cross-correlations of different aaRSs according to their shared CPs. Self correlations were left out for the purpose of clearer presentation. (TIF) [file pone.0020361.s001.tif]

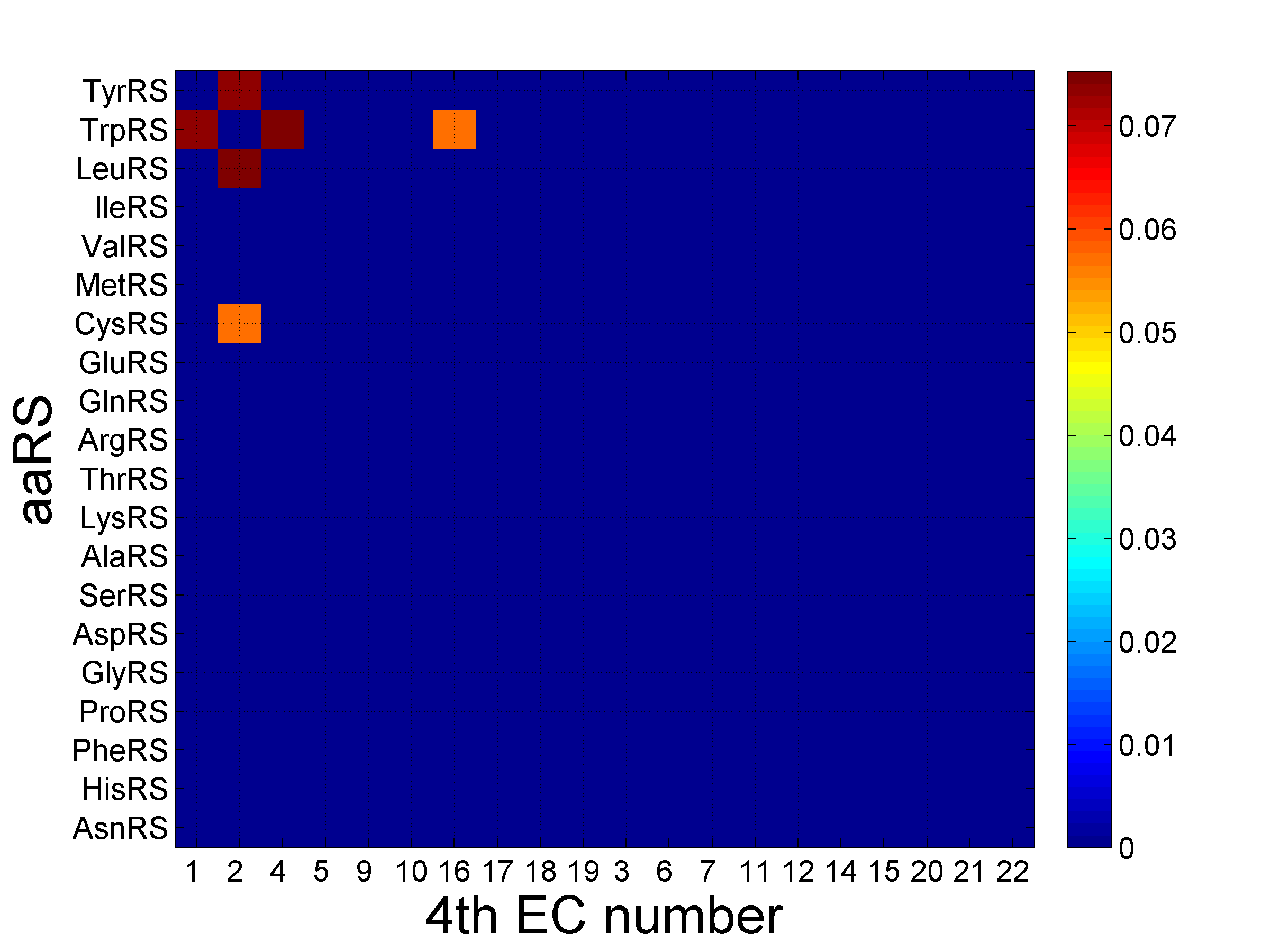

Supplement: Figure S2 — Heat map of Pearson cross-correlations of different aaRSs according to their shared CPs in Bacteria. Self correlations were left out for the purpose of clearer presentation. (TIF) [file pone.0020361.s002.tif]

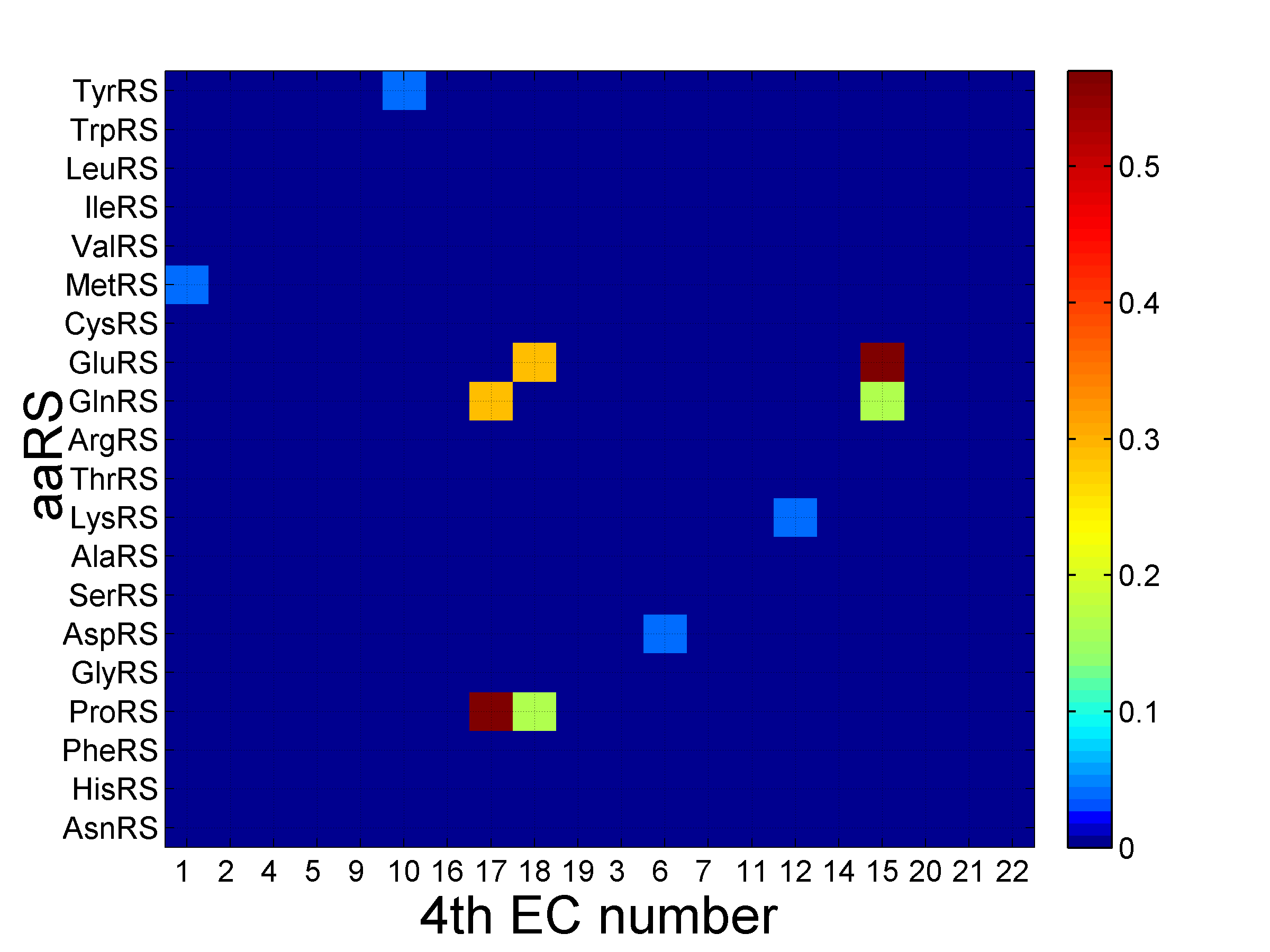

Supplement: Figure S3 — Heat map of Pearson cross-correlations of different aaRSs according to their shared CPs in Eukarya. Self correlations were left out for the purpose of clearer presentation. (TIF) [file pone.0020361.s003.tif]

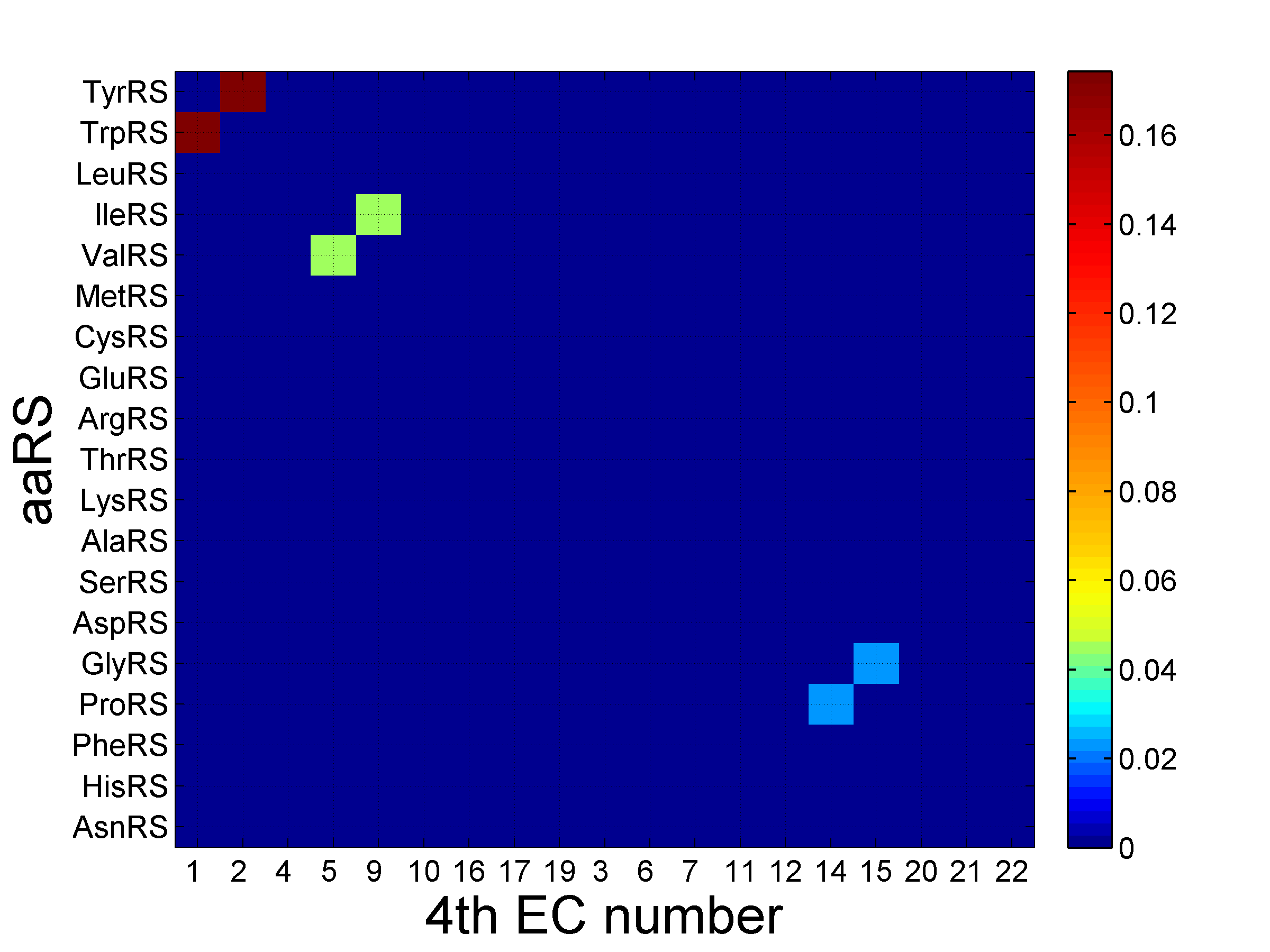

Supplement: Figure S4 — Heat map of Pearson cross-correlations of different aaRSs according to their shared CPs in Archaea. Self correlations were left out for the purpose of clearer presentation. (TIF) [file pone.0020361.s004.tif]

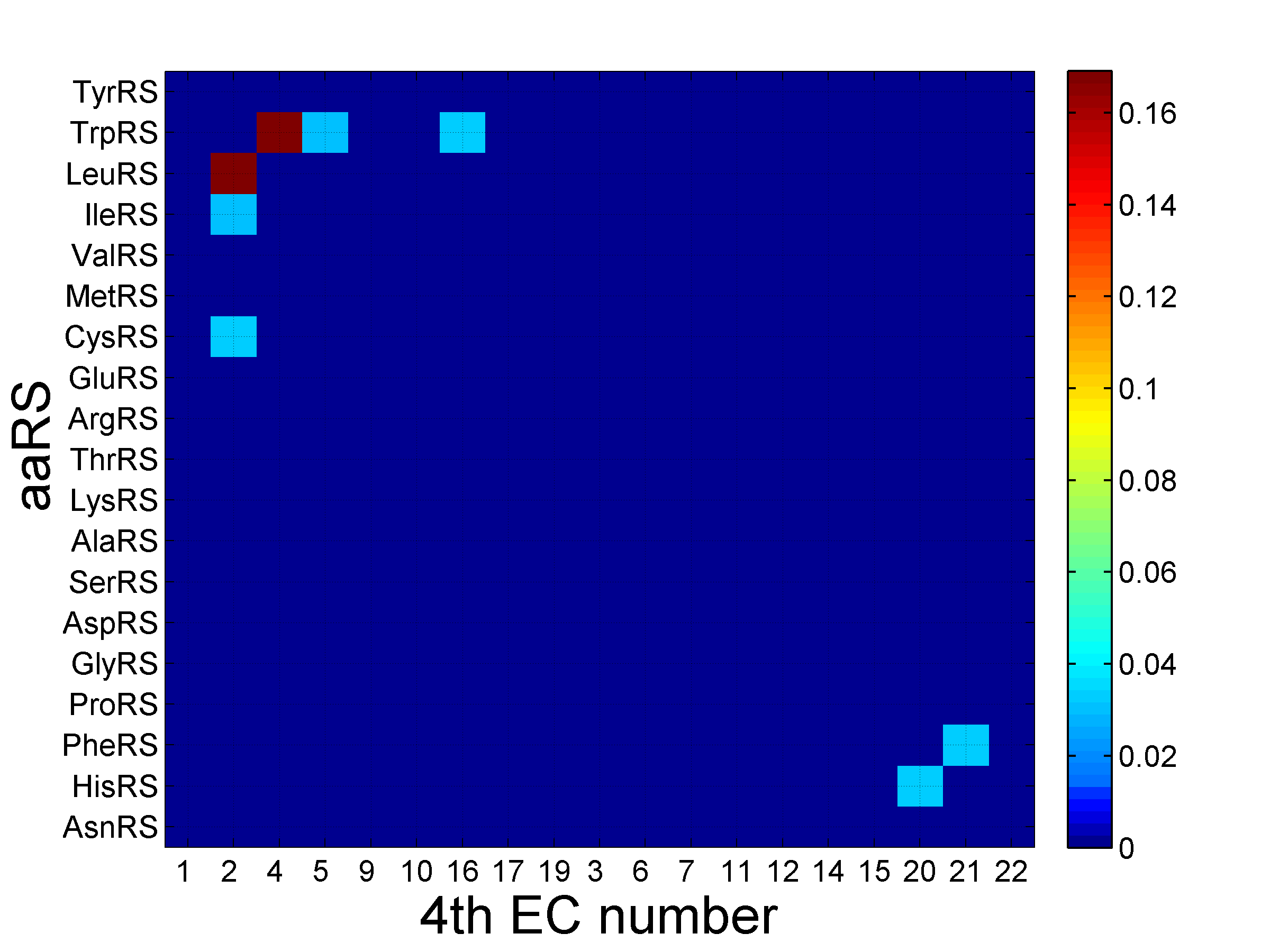

Supplement: Figure S5 — Heat map of Pearson cross-correlations of different aaRSs according to their shared CPs in Mitochondria. Self correlations were left out for the purpose of clearer presentation. (TIF) [file pone.0020361.s005.tif]

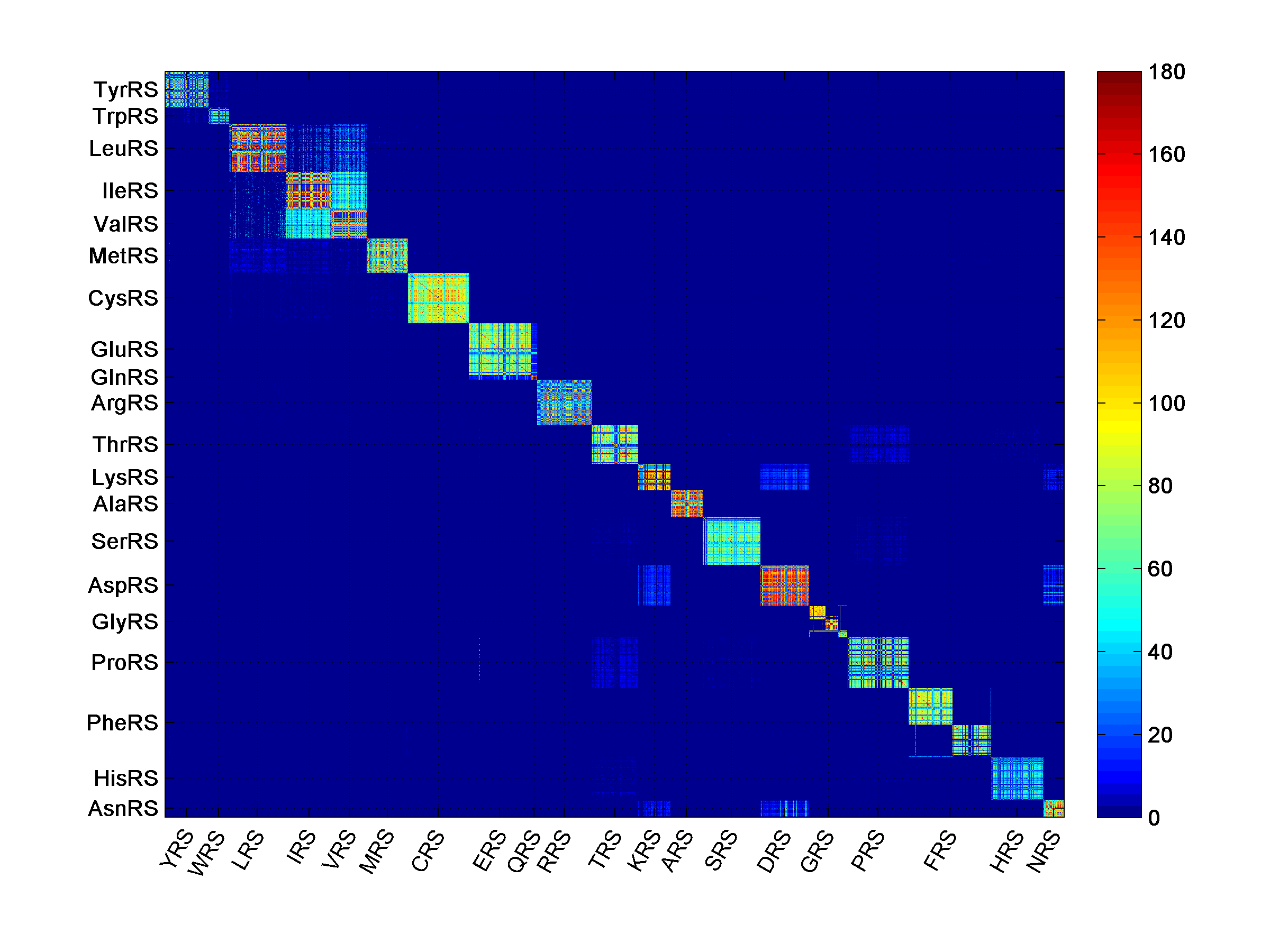

Supplement: Figure S6 — BLAST Similarities between different aaRSs using PFAM domains. E-values> 0.01 are in blue. (TIF) [file pone.0020361.s006.tif]

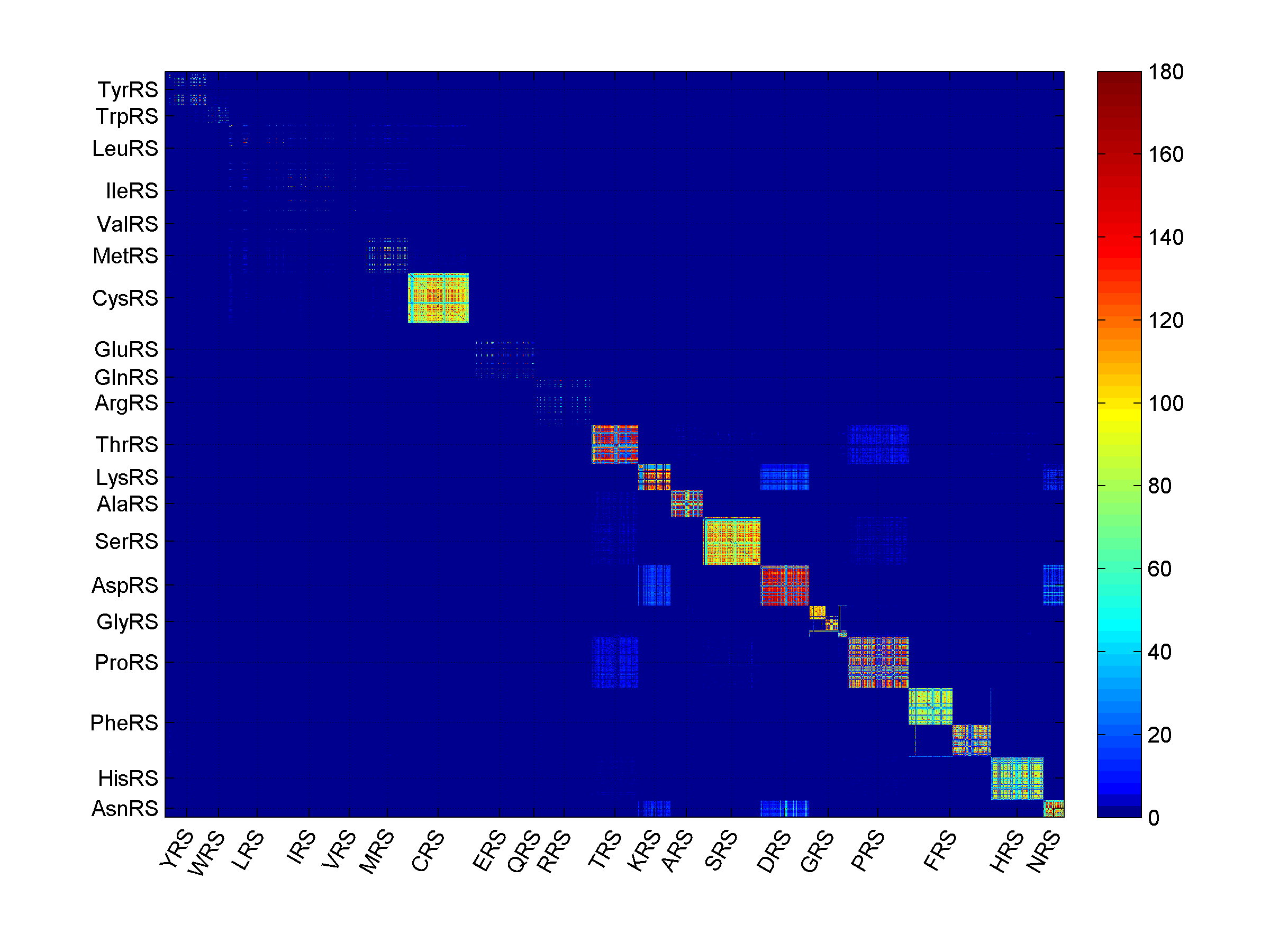

Supplement: Figure S7 — BLAST Similarities between different aaRSs using PROSITE domains. E-values>0.01 are in blue. (TIF) [file pone.0020361.s007.tif]

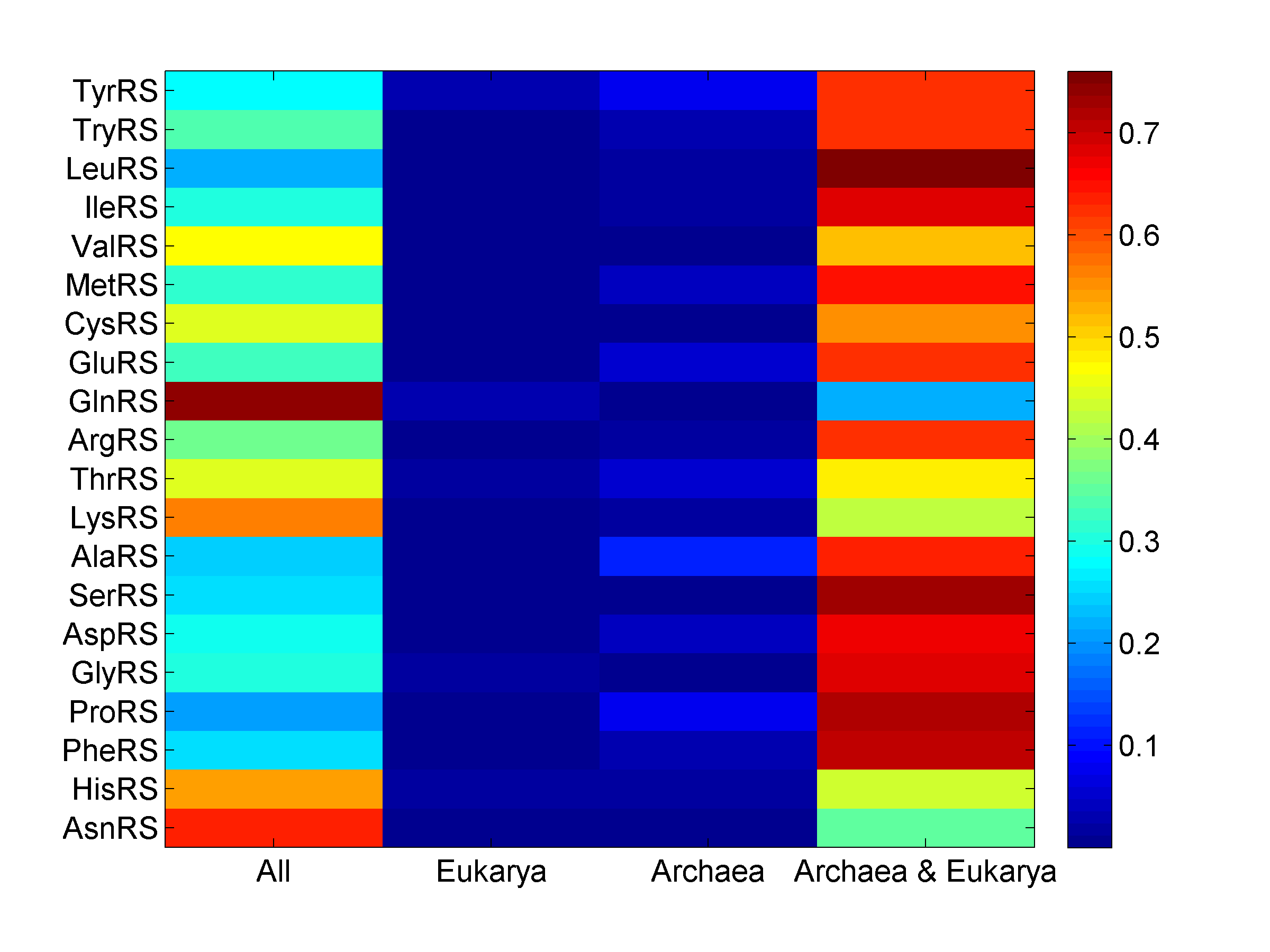

Supplement: Figure S8 — Distribution of different aaRS according to the CPs appearing in all 3 kingdoms together (All), in Bacteria and Eukarya together (excluding Archaea) and in Eukarya and Archaea kingdoms exclusively. (TIF) [file pone.0020361.s008.tif]
