# Supplementary material for: Common Peptides Study of Aminoacyl-tRNA Synthetases
Source: PLoS One. 2011 May 27;6(5):e20361. doi: 10.1371/journal.pone.0020361 (PMC3103580; doi:10.1371/journal.pone.0020361)
Supplement: Table S1 — Occurrence of frequent CPs in aaRSs on sequences with a resolved structure. (PDF) [file pone.0020361.s009.pdf]

Table S1. Occurrence of frequent CPs in aaRSs on sequences with a resolved structure

CP	PDB ID	Uniprot ID	Known binding site
GFGLG	1B8A	SYD_PYRKO	Adenosine Triphosphate
PPHGG	1B8A	SYD_PYRKO	Adenosine Triphosphate *
EVETP	1BBU	SYK1_ECOLI	-
FRNEG	1BBU	SYK1_ECOLI	Lysine
GIGIDR	1BBU	SYK1_ECOLI	Lysine
HNPEF	1BBU	SYK1_ECOLI	Lysine *
DDTNP	1EUQ	SYQ_ECOLI	5'-O-[N-(L-Glutaminyl)-Sulfamoyl]Adenosine
DPRMPT	1EUQ	SYQ_ECOLI	-
GVIHW	1EUQ	SYQ_ECOLI	tRNA binding region
LRAKI	1EUQ	SYQ_ECOLI	tRNA binding region *
SKRKL	1EUQ	SYQ_ECOLI	5'-O-[N-(L-Glutaminyl)-Sulfamoyl]Adenosine
TRFPP	1EUQ	SYQ_ECOLI	5'-O-[N-(L-Glutaminyl)-Sulfamoyl]Adenosine
WDDPR	1EUQ	SYQ_ECOLI	-
YDRLF	1EUQ	SYQ_ECOLI	-
FIGKD	1F4L	SYM_PYRAB	Methionine
PYANG	1F4L	SYM_PYRAB	Methionine
AADIL	1I6K	SYW_BACST	Tryptophanyl-5'Amp
GGRYD	1KMN	SYH_ECOLI	Adenosine Triphosphate
LDLRR	1WYD	SYD_SULTO	-
GEIIGGS	1X54	SYN_PYRHO	4-Amino-1,4-Dioxobutan-2-Aminium Adenosine- 5'-Monophosphate
GFGLG	1X54	SYN_PYRHO	4-Amino-1,4-Dioxobutan-2-Aminium Adenosine- 5'-Monophosphate
GGSQRE	1X54	SYN_PYRHO	4-Amino-1,4-Dioxobutan-2-Aminium Adenosine- 5'-Monophosphate
KAFYM	1X54	SYN_PYRHO	4-Amino-1,4-Dioxobutan-2-Aminium Adenosine- 5'-Monophosphate
LDLRR	1X54	SYN_PYRHO	(4S)-2-Methyl-2,4-Pentanediol *
CGGTH	1YFR	SYA_AQUAE	-
DTGMG	1YFR	SYA_AQUAE	Adenosine Triphosphate
FEMLGN	1YFR	SYA_AQUAE	Adenosine Triphosphate
GDYFK	1YFR	SYA_AQUAE	-
GMGLE	1YFR	SYA_AQUAE	Adenosine Triphosphate
GPCGP	1YFR	SYA_AQUAE	Magnesium Ion *
GRGYV	1YFR	SYA_AQUAE	-
HHTFF	1YFR	SYA_AQUAE	Adenosine Triphosphate
LRFDF	1YFR	SYA_AQUAE	-
SFGDY	1YFR	SYA_AQUAE	Adenosine Triphosphate *
TYGFP	1YFR	SYA_AQUAE	-
DFQAR	2DQ3	SYS_AQUAE	5'-O-(N-(L-Seryl)-Sulfamoyl)Adenosine *
EISSCS	2DQ3	SYS_AQUAE	5'-O-(N-(L-Seryl)-Sulfamoyl)Adenosine
FQARR	2DQ3	SYS_AQUAE	5'-O-(N-(L-Seryl)-Sulfamoyl)Adenosine *
LNGSG	2DQ3	SYS_AQUAE	5'-O-(N-(L-Seryl)-Sulfamoyl)Adenosine
NGSGLA	2DQ3	SYS_AQUAE	5'-O-(N-(L-Seryl)-Sulfamoyl)Adenosine
PTAEV	2DQ3	SYS_AQUAE	5'-O-(N-(L-Seryl)-Sulfamoyl)Adenosine
QLPKF	2DQ3	SYS_AQUAE	-
REISS	2DQ3	SYS_AQUAE	5'-O-(N-(L-Seryl)-Sulfamoyl)Adenosine
TAEVP	2DQ3	SYS_AQUAE	5'-O-(N-(L-Seryl)-Sulfamoyl)Adenosine
TLNGS	2DQ3	SYS_AQUAE	5'-O-(N-(L-Seryl)-Sulfamoyl)Adenosine

VHTLN	2DQ3	SYS_AQUAE	5'-O-(N-(L-Seryl)-Sulfamoyl)Adenosine
MGCYG	2J3L	SYP_ENTFA	'5'-O-(N-(L-Prolyl)-Sulfamoyl)Adenosine
PTHEE	2J3L	SYP_ENTFA	'5'-O-(N-(L-Prolyl)-Sulfamoyl)Adenosine
LRIEDT	2O5R	SYE1_THEMA	-
PSPTG	2O5R	SYE1_THEMA	Chloride Ion
RFAPSP	2O5R	SYE1_THEMA	Chloride Ion
RIEDTD	2O5R	SYE1_THEMA	-

* The CP is distant by at most 3 residues from the site.
